# Mechanistic insights into pH-dependent H_2_ photoproduction in bisulfite-treated *Chlamydomonas* cells

**DOI:** 10.1186/s13068-020-01704-0

**Published:** 2020-04-06

**Authors:** Lanzhen Wei, Baoqiang Fan, Jing Yi, Tianqun Xie, Kun Liu, Weimin Ma

**Affiliations:** Shanghai Key Laboratory of Plant Molecular Sciences, College of Life Sciences, Shanghai Normal University, Guilin Road 100, Shanghai, 200234 China

**Keywords:** pH, Bisulfite, Sulfite, H_2_ photoproduction, *Chlamydomonas reinhardtii*

## Abstract

**Background:**

Bisulfite addition is an important H_2_ photoproduction strategy that removes O_2_ and activates hydrogenase. The pH values of cell cultures can change the ratio of bisulfite to sulfite, which may affect H_2_ photoproduction. However, little is known regarding the pH effect of bisulfite addition on H_2_ photoproduction and relevant underlying mechanism.

**Results:**

Here, changes in H_2_ photoproduction with different initial extracellular pH values showed a parabolic distribution and a pH of 8.0 is an optimal value for H_2_ photoproduction in *Chlamydomonas reinhardtii* cells treated with bisulfite. Compared to the growth pH (pH 7.3), increased photoproduction of H_2_ at this optimal pH was primarily caused by a relatively high residual activity of photosystem II (PSII), which provides a relatively plentiful source of electrons for H_2_ photoproduction. Such increased H_2_ photoproduction was most likely a result of decreased the ratio of bisulfite to sulfite, consistent with the result that the toxicity of bisulfite on PSII was much more than that of sulfite. This possibility was corroborated by the result that treatment with a combination of 7 mM bisulfite and 6 mM sulfite further enhanced H_2_ photoproduction compared with 13 mM bisulfite alone.

**Conclusions:**

Collectively, our findings provide novel mechanistic insights into pH-dependent H_2_ photoproduction in *C. reinhardtii* cells treated with bisulfite, and demonstrate that sulfite addition is another important strategy for H_2_ photoproduction, just like bisulfite addition.

## Background

Increased awareness of fossil fuel depletion and global warming has led to extensive efforts to develop clean and renewable energy sources (for reviews, see [1, 2]). Molecular hydrogen (H_2_) is considered to be one of the most promising future energy sources because its combustion only produces H_2_O as a waste product [3, 4]. *Chlamydomonas reinhardtii*, a unicellular green alga, has been recognized as an ideal candidate among eukaryotes for photobiological H_2_ production because its [Fe–Fe]-hydrogenase (H_2_ase) exhibits a higher specific activity than exhibited by [Ni–Fe]-H_2_ases in some other microorganisms [5, 6]. Under natural conditions, however, *C. reinhardtii* only produces H_2_ under anaerobic conditions because its [Fe–Fe]-H_2_ase is extremely sensitive to oxygen (O_2_) [7]. As a consequence, numerous strategies are developed to activate [Fe–Fe]-H_2_ase in *C. reinhardtii* for efficient and sustainable H_2_ photoproduction (for recent reviews, see [8–10]), including (1) developing the O_2_-tolerant [Fe–Fe]-H_2_ase [11, 12]; and (2) decreasing the O_2_ content around [Fe–Fe]-H_2_ase [13–19].

Nearly one decade ago, we also developed an alternative H_2_ photoproduction strategy that treatment of *C. reinhardtii* cells with bisulfite (NaHSO_3_) activates H_2_ase by decreasing the O_2_ levels in those cells [20]. Such decrease was found to be a result of efficient reaction of bisulfite with superoxide anion under sufficient light conditions [21]. We further found that regardless of an approximately 200-fold increase in H_2_ photoproduction was induced by this strategy in *C. reinhardtii* cells [20], but its yield was significantly suppressed by impaired PSII [22], an electron source for H_2_ photoproduction [23–25]. Thus, it is logic to hypothesize that this strategy has a great potential for enhancing the yield of H_2_ photoproduction in *C. reinhardtii* cells through improving PSII activity.

Numerous studies have reported that optimal pH values are important for efficient production of H_2_ in cyanobacteria [26–28] and green algae [29, 30]. For example, in sulfur-deprived *Chlamydomonas* cells, a pH of 7.7 is an optimal value to lead to maximum H_2_ photoproduction, which is closely associated with residual PSII activity but less with starch and protein degradation [29]. In addition, we noticed that SO_2_ derivatives at least contain bisulfite and sulfite (Na_2_SO_3_), and pH values can change their ratio in the cell cultures [31]. Moreover, the toxicity of bisulfite to the growth of algal cells was much more than that of sulfite [32, 33]. Collectively, we hypothesized that pH is able to change H_2_ photoproduction via affecting the ratio of bisulfite to sulfite in the cell cultures. However, little is known regarding the pH effect of bisulfite addition on the yield of H_2_ photoproduction and relevant underlying mechanism.

To investigate the pH effect of bisulfite addition on H_2_ photoproduction and relevant underlying mechanism, we first examined the effects of different initial extracellular pH values on H_2_ photoproduction in NaHSO_3_-treated *C. reinhardtii* cells. We then assessed the degree to which H_2_ photoproduction increased at the optimal pH and indicated the possible action target site that was associated with this increased H_2_ production. Finally, we compared the residual activity of PSII and the yield of H_2_ photoproduction under conditions of bisulfite and sulfite both with that under conditions of bisulfite alone.

## Results

### Effect of initial extracellular pH on H_2_ photoproduction in NaHSO_3_-treated *C. reinhardtii* cells

**Fig. 1:**
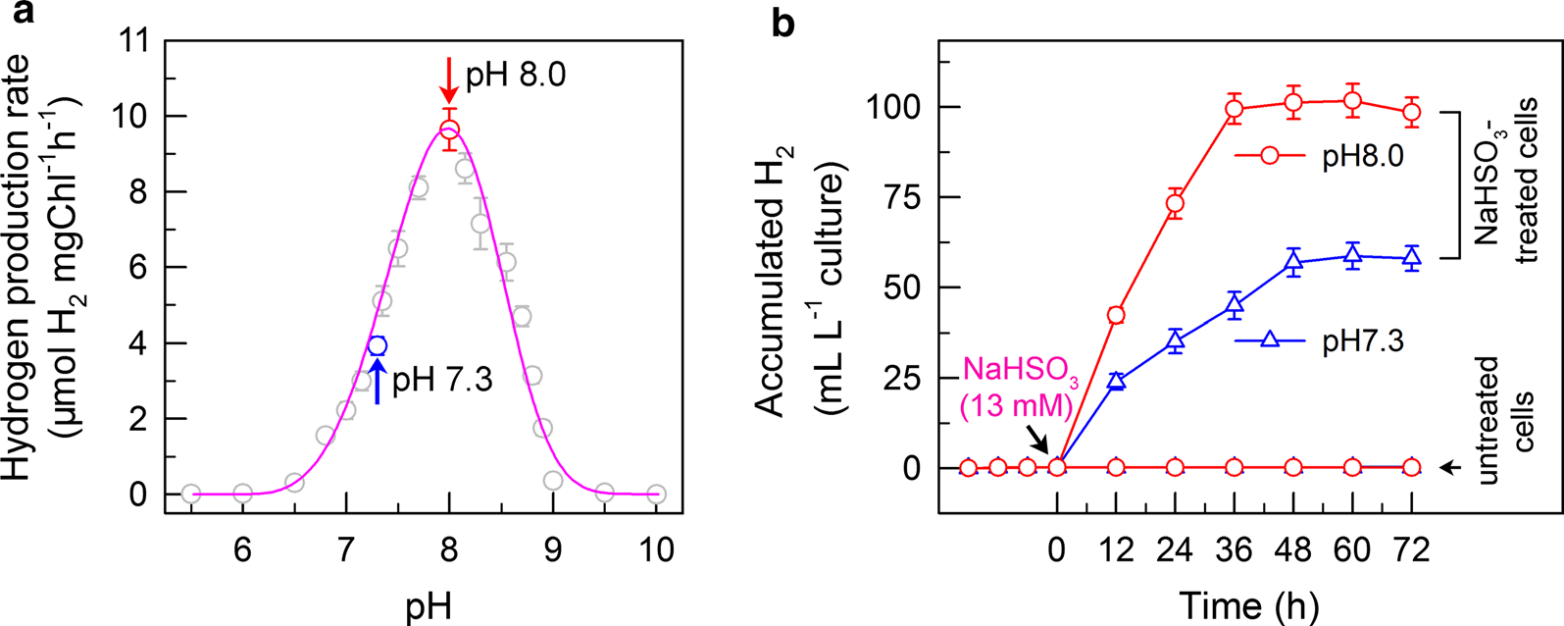
Treatment with optimal pH significantly increases the yield of H_2_ photoproduction in *C. reinhardtii* cells treated with NaHSO_3_. **a** Effect of treatment with different initial extracellular pH values on the rate of H_2_ photoproduction in *C. reinhardtii* cells treated with NaHSO_3_. The rate of H_2_ photoproduction by *C. reinhardtii* cells was calculated within 12 h after NaHSO_3_ addition. **b** Effect of treatment with optimal and growth pH values on the yield of H_2_ photoproduction in *C. reinhardtii* cells treated with NaHSO_3_. Red and blue arrows in **a** indicate optimal pH (pH 8.0) and growth pH (pH 7.3), respectively. Values are mean ± SD (*n* = 5)

Changes in the rates of H_2_ photoproduction with different initial extracellular pH values showed a parabolic distribution (Fig. 1a). In specific, the maximum rate of H_2_ photoproduction was observed to occur at pH 8.0 (see red arrow in Fig. 1a), and any increase or decrease in initial extracellular pH resulted in a lower rate of H_2_ photoproduction (Fig. 1a). This finding indicates that H_2_ photoproduction is enhanced at moderate pH levels, and that a pH of 8.0 is an optimal value to result in maximum H_2_ photoproduction in NaHSO_3_-treated cells of *C. reinhardtii*.

### The yield of H_2_ photoproduction at pH 8.0 is greatly enhanced in NaHSO_3_-treated *C. reinhardtii* cells

Levels of H_2_ increased immediately after treatment with NaHSO_3_ (see black arrow in Fig. 1b) and remained high, whereas the H_2_ level was almost unchanged and remained low in the untreated cells, regardless of cellular incubation at either pH 8.0 or pH 7.3 (growth pH; see blue arrow in Fig. 1a). This finding is in agreement with the results reported in previous studies [20, 34]. In NaHSO_3_-treated cells, the yield of H_2_ photoproduction at pH 8.0 can be further enhanced when compared to the pH 7.3 (Fig. 1b). In specific, the H_2_ level in NaHSO_3_-treated cells incubated at pH 8.0 was approximately 1.75 times greater than the level observed at pH 7.3 (Fig. 1b). We therefore conclude that the yield of H_2_ photoproduction at pH 8.0 is significantly increased under NaHSO_3_ addition conditions.

### An anaerobic environment established by NaHSO_3_ at pH 8.0 is relatively slow but H_2_ase activity is more strongly stimulated

**Fig. 2:**
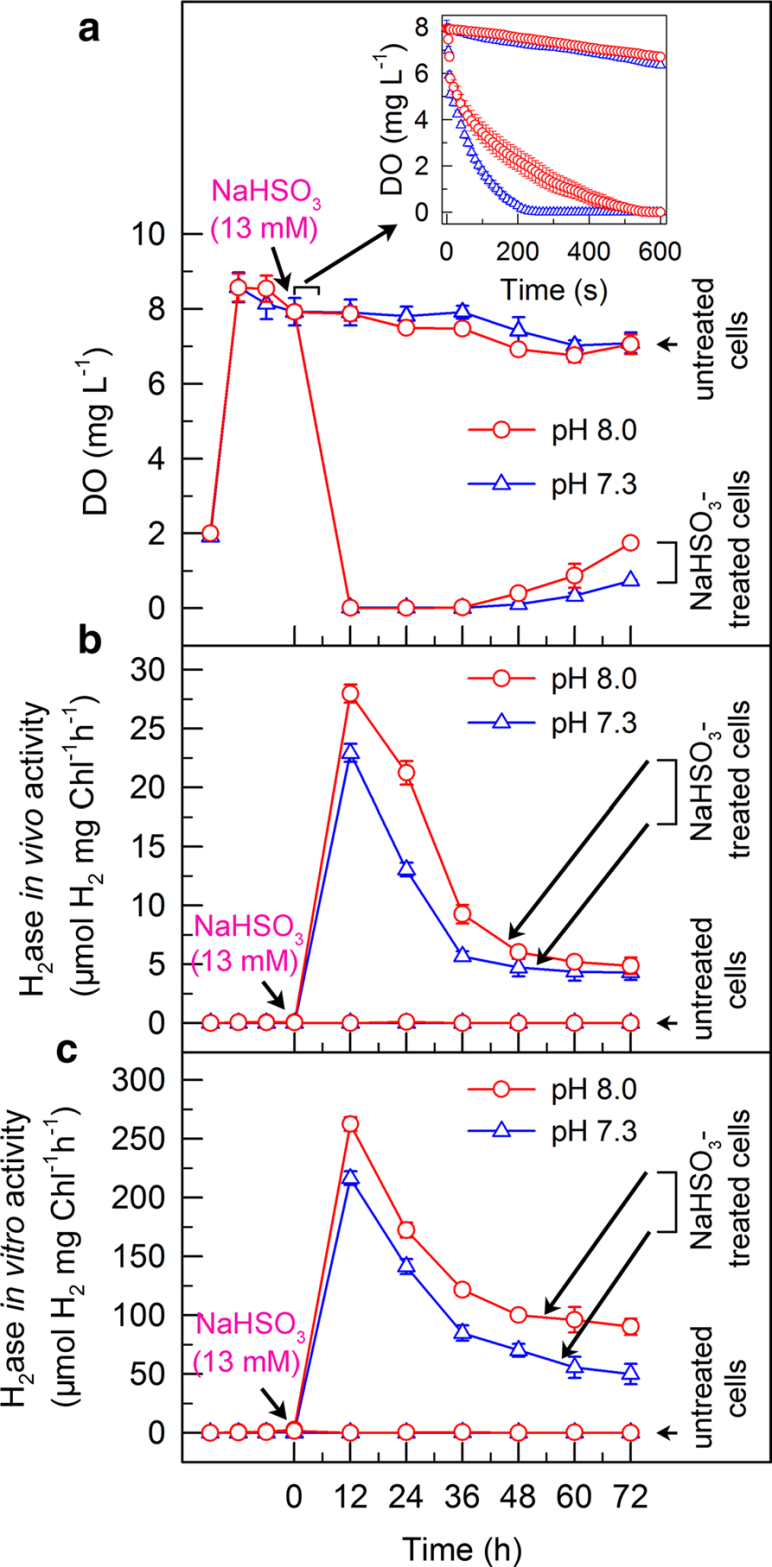
Treatment with optimal pH decreases dissolved oxygen (DO) content (**a**) and consequently increases in vivo (**b**) and in vitro (**c**) H_2_ase activity in *C. reinhardtii* cells treated with NaHSO_3_. Values are mean ± SD (*n* = 5)

To elucidate the mechanism by which H_2_ photoproduction increased at pH 8.0 in NaHSO_3_-treated cells, we monitored dissolved O_2_ content alongside H_2_ase activity. Our results indicated that treatment with NaHSO_3_ rapidly creates an anaerobic environment in the cell cultures (Fig. 2a), which stimulates H_2_ase activity regardless of initial extracellular pH (Fig. 2b, c). Surprisingly, compared to the pH 7.3, an anaerobic environment generated by NaHSO_3_ addition at pH 8.0 was relatively slow (insert in Fig. 2a), but H_2_ase activity was more strongly increased (Fig. 2b, c). This result indicates that levels of H_2_ increased at pH 8.0 are independent of O_2_ content in the background of bisulfite addition. Future studies are required to clarify the interrelationship of the duration of H_2_ production at different initial extracellular pH values with O_2_ content in the bisulfite addition strategy.

Recently, we have demonstrated that 13 mM of NaHSO_3_ as an optimal concentration for H_2_ photoproduction in *C. reinhardtii* [20] can remove O_2_ efficiently in intact cells through a reaction of bisulfite with superoxide anion radicals produced at the acceptor side of PSI, especially under sufficient light conditions [21]. A similar process has been previously reported to operate in spinach chloroplasts [35] and tobacco thylakoids [36]. We named the mechanism of removal of O_2_ molecules as bisulfite photooxidation, since operation of this mechanism requires light irradiation and oxidation reaction of bisulfite. Here, we noticed that a photooxidation level of bisulfite at pH 8.0 was lower than that at pH 7.3 (Additional file 1: Figure S1a), consistent with the result that establishment of an anaerobic environment at pH 8.0 was slower than that at pH 7.3 (insert in Fig. 2a). This is most likely the result of less superoxide anion radicals at pH 8.0 and more superoxide anion radicals at pH 7.3 (Additional file 1: Figure S1b).

### A residual activity of electron source maintained at pH 8.0 is relatively high under NaHSO_3_ addition conditions

**Fig. 3:**
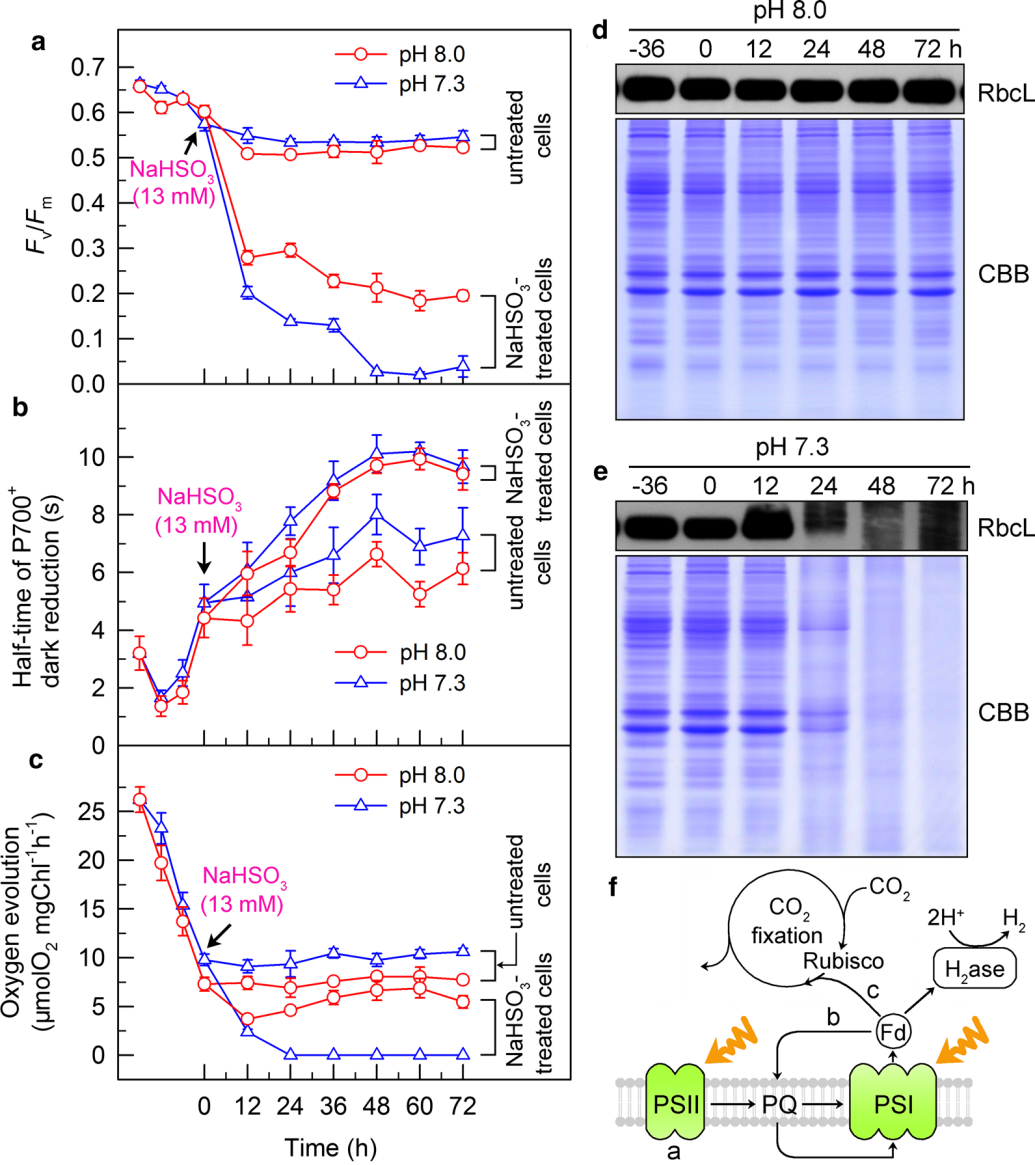
Treatment with optimal pH alleviates the inhibitory effects of NaHSO_3_ addition on PSII activity (**a**), cyclic electron transfer around PSI (**b**), and CO_2_ assimilation (**c**–**e**) in *C. reinhardtii* cells. **a** PSII activity was evaluated by calculated *F*_v_/*F*_m_ values. **b** The rate of cyclic electron transfer around PSI was judged by half-time of P700^+^ dark reduction. **c**–**e** Activity of CO_2_ assimilation was assessed by photosynthetic production of O_2_ with NaHCO_3_ as an artificial electron acceptor (**c**), Rubisco expression levels (**d**, **e**). Coomassie Brilliant Blue (CBB) staining profiles of total proteins from untreated cells and NaHSO_3_-treated cells (including designated time points) at pH 8.0 (**d**) and pH 7.3 (**e**) and their immunoblotting using the antibody against RbcL. A 20-µg aliquot of total protein was loaded onto each lane. Values are mean ± SD (*n* = 5). **f** Schematic model representing the relationship of H_2_ photoproduction with its electron source and alternative electron sinks

To assess whether the enhanced production of H_2_ at pH 8.0 is driven by a relatively plentiful source of electrons (see a in Fig. 3f), we monitored the activity of PSII in NaHSO_3_-treated cells incubated at different pH values. The results revealed that NaHSO_3_ addition impaired PSII activity regardless of initial extracellular pH level [see the calculated values of maximum quantum yield of PSII (*F*_v_/*F*_m_); Fig. 3a]. A residual activity of PSII maintained at pH 8.0, however, is relatively high in comparison to the pH 7.3, under NaHSO_3_ addition conditions, consistent with the results that the amount of superoxide anion radicals at pH 8.0 was less than that at pH 7.3 (Additional file 1: Figure S1b). This finding strongly suggests that the increased production of H_2_ at pH 8.0 is primarily driven by the maintenance of a relatively high residual activity of PSII at this pH, which provides a relatively plentiful source of electrons for H_2_ photoproduction.

### The activities of alternative electron sinks for H_2_ photoproduction at pH 8.0 are increased under NaHSO_3_ treatment conditions

Increased H_2_ production at pH 8.0 may also be driven by the activity of alternative electron sinks for H_2_ photoproduction (see b and c in Fig. 3f). To assess the likelihood of this possibility, we monitored the activity of cyclic electron transport around photosystem I (PSI CET) and also assessed the activity of CO_2_ fixation. Our results showed that treatment with NaHSO_3_ greatly reduced the activity of PSI CET in cells incubated at different pH values, as judged by the half-time of P700^+^ re-reduction in darkness (Fig. 3b). Importantly, however, the activity of PSI CET at pH 8.0 is slightly increased when compared to the pH 7.3 (Fig. 3b). We further found that treatment with NaHSO_3_ significantly decreased the activity of CO_2_ fixation in cells incubated at different pH values, as estimated by the photosynthetic production of O_2_ with NaHCO_3_ as an artificial electron acceptor (Fig. 3c). As expected, under NaHSO_3_ addition conditions, a residual activity of CO_2_ assimilation maintained at pH 8.0 was relatively higher than that at pH 7.3 (Fig. 3c). This difference was reinforced by the results of Rubisco accumulation in cells. As deduced from the accumulation levels of Rubisco large (RbcL) subunit in cells, we observed that the expression levels of Rubisco were always maintained at a relatively high level under conditions of pH 8.0 but significantly decreased under conditions of pH 7.3, especially after 12 h, with the prolonged time of NaHSO_3_ addition (Fig. 3d, e). Collectively, it appears plausible that at least the two alternative electron sinks for H_2_ photoproduction are not responsible for the increased H_2_ photoproduction observed at pH 8.0.

**Fig. 4:**
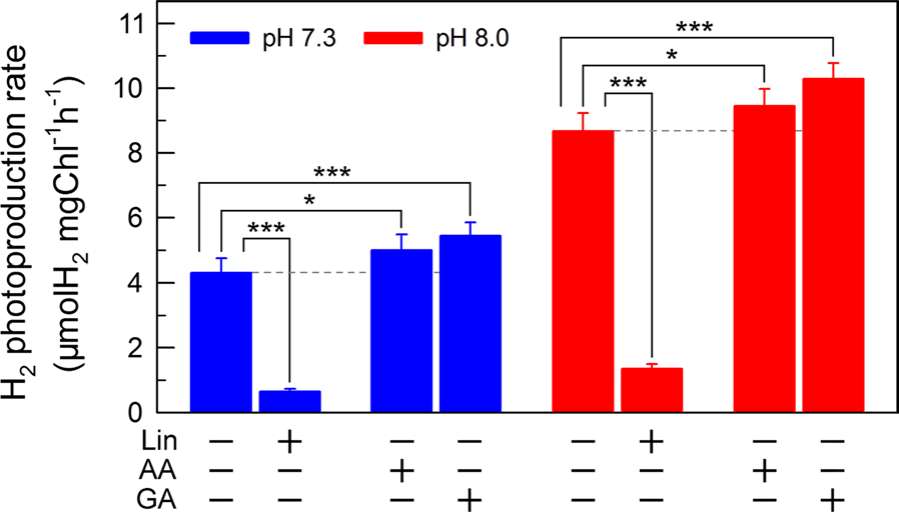
The yield of H_2_ photoproduction in NaHSO_3_-treated *C. reinhardtii* cells with multiple inhibitors. After cells were statically pre-cultured under continuous illumination of 200 µE m^−2 ^s^−1^ for 36 h, NaHSO_3_ (13 mM) and several inhibitors, lincomycin (Lin; 5 mM), antimycin A (AA; 10 µM), and glycolaldehyde (GA; 2 mM), were added to the serum bottles, respectively. Values are mean ± SD (*n* = 5). *p < 0.05; ***p < 0.001

If this possibility is true, an increase in H_2_ photoproduction caused by impaired the activity of either PSI CET or CO_2_ assimilation will be higher in cells incubated at pH 8.0 than that at pH 7.3. The results shown in Fig. 4 support our hypothesis that the increase in H_2_ photoproduction was slightly higher in cells incubated at pH 8.0 than that at pH 7.3 in the presence of either antimycin A (AA) that specifically inhibits the PSI CET activity [37] or glycolaldehyde (GA) that disrupts the Calvin–Benson cycle activity via inhibiting the phosphoribulokinase [38]. Collectively, we may conclude that in the anaerobic background, increased residual PSII activity can significantly enhance the yield of H_2_ photoproduction in *C. reinhardtii*.

If this conclusion is true, impaired PSII activity in an anaerobic environment created by NaHSO_3_ addition will inevitably decrease the yield of H_2_ photoproduction in *C. reinhardtii* at a significant level, especially at pH 8.0. As expected, the H_2_ photoproduction rate was significantly decreased in the presence of lincomycin (Lin), which impairs the PSII activity through inhibiting the D1 protein synthesis [39], regardless of either optimal or growth pH (Fig. 4). Importantly, such decrease was much more in cells incubated at pH 8.0 than that at pH 7.3 (Fig. 4). These results greatly consolidate our conclusion that increased residual PSII activity in an anaerobic environment is an efficient strategy to improve H_2_ photoproduction in *C. reinhardtii* and the optimal pH is a case study in this strategy.

### Treatment with a combination of bisulfite and sulfite enhances H_2_ photoproduction further

With the pH values increased, a ratio of bisulfite to sulfite decreased and the toxicity of bisulfite–sulfite on photosynthesis also decreased [31], consistent with the results that the toxicity of sulfite on photosynthesis was weaker than that of bisulfite [32, 33]. Therefore, it is logical to hypothesize that a combination of bisulfite and sulfite can improve the yield of H_2_ photoproduction by increasing residual PSII activity.

**Fig. 5:**
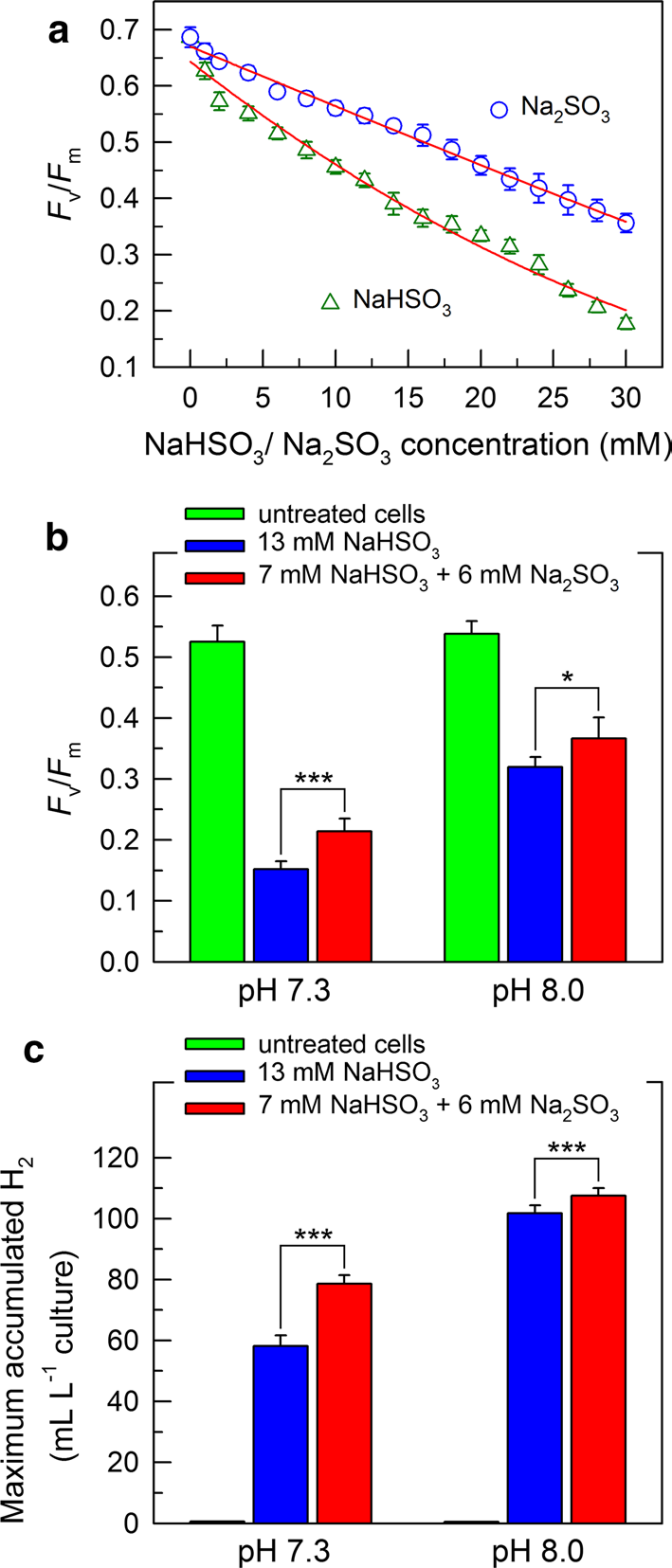
Treatment with a combination of NaHSO_3_ and Na_2_SO_3_ significantly enhances the yield of H_2_ photoproduction in *C. reinhardtii* cells. **a** Effect of treatment with different concentrations of either NaHSO_3_ or Na_2_SO_3_ on PSII activity. The Chl concentration was adjusted to 10 µg mL^−1^ and under the intensity of 200 μE m^−2^ s^−1^, NaHSO_3_ or Na_2_SO_3_ with different concentrations was added to the cell cultures for 5 min before measurement. Subsequently, PSII activity was immediately measured using a Dual-PAM-100 monitoring system. **b**, **c** Effect of treatment with a combination of 7 mM NaHSO_3_ and 6 mM Na_2_SO_3_ on PSII activity (**b**) and H_2_ photoproduction (**c**) at different pH values. PSII activity was evaluated by calculated *F*_v_/*F*_m_ values. Values are mean ± SD (*n* = 5). *p < 0.05; ***p < 0.001

To test this idea, we compared the inhibitory effects of bisulfite and sulfite on PSII activity. Clearly, the toxicity of bisulfite on PSII was greater than that of sulfite as deduced from the changes in *F*_v_/*F*_m_ values under conditions of different concentrations of bisulfite or sulfite (Fig. 5a). As a consequence, a combination of bisulfite and sulfite with a total of 13 mM alleviated their toxicity on PSII in comparison to 13 mM of bisulfite alone, regardless of cells incubated at pH 8.0 or pH 7.3 (Fig. 5b). As expected, the combination improved the yield of H_2_ photoproduction regardless of pH 8.0 or pH 7.3 (Fig. 5c). Furthermore, the degree of such alleviation of PSII at pH 7.3 was more evident than that at pH 8.0 (Fig. 5b). This may be because the ratio of bisulfite to sulfite changed at pH 7.3 was more than that at pH 8.0 after 13 mM of NaHSO_3_ was added to the cultures. Consistently, the degree of H_2_ photoproduction improved at pH 7.3 was greater than that at pH 8.0 (Fig. 5c). Regardless of their differences, treatment with a combination of bisulfite and sulfite can further enhance the yield of H_2_ photoproduction in *C. reinhardtii* cells.

## Discussion

Whether bisulfite addition functions in photosynthetic O_2_ evolution or photosynthetic H_2_ evolution depends on its concentrations: bisulfite in a low amount improves photosynthetic O_2_ evolution [34, 40], but in a moderate amount can significantly promote photosynthetic H_2_ evolution [20, 34]. It has been demonstrated that a low amount (100 μM) of bisulfite improves photosynthesis by increasing cyclic photophosphorylation and optimizing ATP/NADPH ratio required for the Calvin–Benson cycle [40]. By contrast, a moderate amount (13 mM) of bisulfite can remove O_2_ efficiently through a reaction of bisulfite with superoxide anion produced at the acceptor side of PSI, especially under sufficient light conditions, and consequently activates H_2_ase and promotes H_2_ photoproduction [21]. Consistent with other H_2_ photoproduction strategies [23–25], the source of electrons for H_2_ photoproduction in our bisulfite addition strategy predominantly, if not totally, comes from water photolysis via PSII [22] (Figs. 3, 4); unfortunately, impaired PSII by bisulfite addition greatly limits the efficient photoproduction of H_2_ in *C. reinhardtii*. Therefore, increased residual PSII activity in an anaerobic environment is an efficient strategy to improve H_2_ photoproduction in *C. reinhardtii* further.

The stepwise bisulfite addition mode of our previous study [22] and the optimal pH of this study are two case studies to improve residual PSII activity for increasing H_2_ photoproduction in NaHSO_3_-treated cells of *C. reinhardtii*. It is easy to understand the reason why the stepwise bisulfite addition mode can alleviate the toxicity of bisulfite on PSII, thereby resulting in an increase in H_2_ photoproduction. In contrast, it is difficult to understand the reason why the optimal pH can alleviate the toxicity of bisulfite on PSII, causing an increase in H_2_ photoproduction. Occasionally, we noticed that treatment with the optimal pH can decrease the ratio of bisulfite to sulfite [31]. It is logical to hypothesize that such decrease can lead to an increase in residual PSII activity, since the toxicity of sulfite on photosynthesis is lower than that of bisulfite [32, 33]. This hypothesis was confirmed by the results of this study (Fig. 5). Collectively, we propose that decreasing the ratio of bisulfite to sulfite by the optimal pH increases residual PSII activity, thereby enhancing the yield of photobiological H_2_ production in *C. reinhardtii* cells.

We also noticed that oxidation of SH groups of the enzymes that constitute the Calvin–Benson cycle, such as glyceraldehyde 3-phosphate dehydrogenase, by bisulfite and sulfite directly or indirectly [41, 42] leads to production of reactive oxygen species (ROS), which suppresses synthesis of the D1 protein during the repair of PSII after photodamage [43] and destabilizes the PSII architecture [44]. Consistent with these findings, the residual Calvin–Benson cycle activity at pH 8.0 is higher and resultant ROS level is lower than that at pH 7.3 (Fig. 3c–e and Additional file 1: Figure S1b). Collectively, the toxicity of bisulfite on PSII is higher than that of sulfite possibly through suppressing more residual Calvin–Benson cycle activity and producing more ROS molecules. However, the mechanism by which bisulfite and sulfite exert their toxicity on PSII remains elusive. Future studies are required to unravel this mechanism and fully understand different effects of bisulfite and sulfite on PSII activity.

Our previous data indicated that, when an initial concentration of bisulfite in stepwise addition mode was equal to or less than 7 mM, the cell suspension cultures did not enter or maintain an anaerobic environment, which evidently suppressed the increase in H_2_ photoproduction in the stepwise addition mode [22]. By comparison, a combination of 7 mM bisulfite and 6 mM sulfite can quickly establish an anaerobic environment (Additional file 1: Figure S2). It appears plausible that sulfite can also react with superoxide anion to remove O_2_ in cells of *C. reinhardtii*. This possibility was verified by the results of increased sulfate with a significant level (Additional file 1: Table S1). As a consequence, sulfite addition can also efficiently promote H_2_ photoproduction via removing O_2_ and activating H_2_ase (Additional file 1: Figure S3a–d and Additional file 1: Table S1) just like bisulfite addition.

**Fig. 6:**
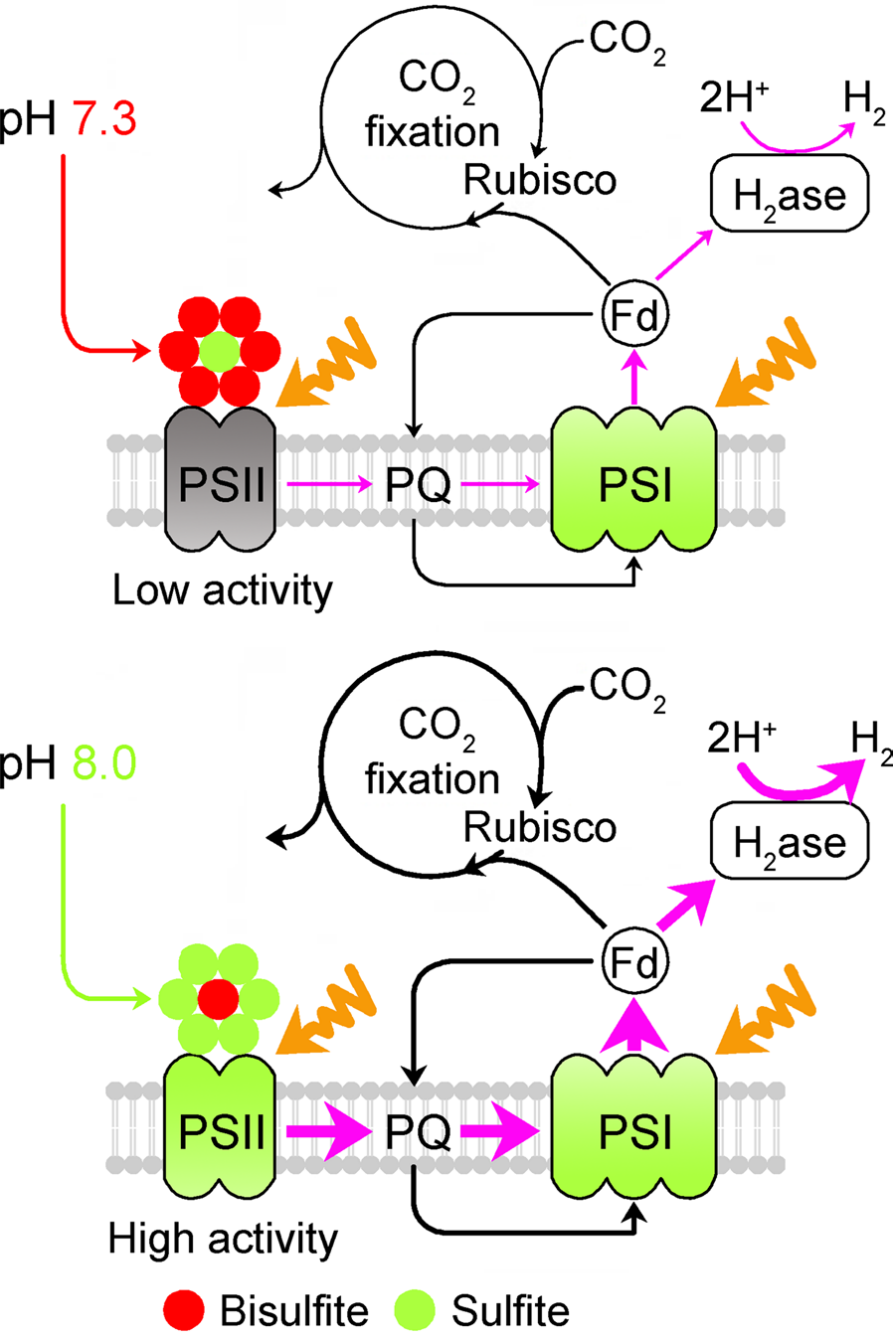
Schematic model representing the mechanism that optimal pH increases the yield of H_2_ photoproduction in NaHSO_3_-treated cells of *C. reinhardtii*. Compared to growth pH (pH 7.3), optimal pH (pH 8.0) decreases the ratio of NaHSO_3_ to Na_2_SO_3_ and consequently alleviates the inhibitory effects of NaHSO_3_ addition on PSII activity, and increases H_2_ photoproduction. The widths of the pink and black arrows indicate the levels of electron source and alternative electron sinks (PSI CET and CO_2_ fixation) for H_2_ photoproduction, respectively

Collectively, our data reported here provide novel mechanistic insights into pH-dependent H_2_ photoproduction in bisulfite-treated cells of *C. reinhardtii*. In this model, addition of bisulfite to the cell cultures incubated at pH 7.3 has a high ratio of bisulfite to sulfite, which significantly suppresses water photolysis via PSII, and leads to a low source of electrons for H_2_ photoproduction (Fig. 6). In contrast, a low ratio of bisulfite to sulfite created by pH 8.0 promotes water photolysis via PSII and maintains a relatively high source of electrons for H_2_ photoproduction (Fig. 6). Compared to the high ratio of bisulfite to sulfite created by pH 7.3, the low ratio of bisulfite to sulfite created by pH 8.0 also slightly increases the activities of two alternative sinks of electrons for H_2_ photoproduction, PSI CET and CO_2_ assimilation (Figs. 3, 6). We thus propose that the yield of H_2_ photoproduction was influenced by pH in *C. reinhardtii* cells mainly through changing the ratio of bisulfite to sulfite and subsequent the level of water photolysis via PSII, an electron source for H_2_ photoproduction.

Although impaired PSII has been improved by the stepwise bisulfite addition mode or the optimal pH or a combination of bisulfite and sulfite, the residual PSII activity is still at a relatively low level. This indicates that there has a great potential to enhance the yield of H_2_ photoproduction in the background of an anaerobic environment created by bisulfite and/or sulfite addition via increasing the residual PSII activity. Therefore, it is very important to identify these potential key targets that suppress the residual PSII activity in the background of bisulfite and/or sulfite addition using forward genetics strategy and to further enhance the yield of H_2_ photoproduction in algal cells in the future.

Alternatively, recent studies have demonstrated that suppression of the Calvin–Benson cycle results in a sustainable and efficient photoproduction of H_2_ in *C. reinhardtii* [25, 45]. Consistent with these findings, an evident suppression of the Calvin–Benson cycle by bisulfite at pH 7.3 causes a longer H_2_ photoproduction (Fig. 1b) and by GA, a Calvin–Benson cycle inhibitor, leads to a more efficient H_2_ photoproduction regardless of cells incubated at pH 8.0 or pH 7.3 (Fig. 4). Collectively, besides the PSII target, the Calvin–Benson cycle should be an alternative target that results in a sustainable and efficient photoproduction of H_2_ in bisulfite-treated algal cells.

## Conclusions

In this study, we have demonstrated that the yield of H_2_ photoproduction is increased by an optimal pH in *C. reinhardtii* cells treated with bisulfite. Our results further revealed that this increased H_2_ photoproduction was mainly caused by the maintenance of a relatively high residual activity of electron source (PSII) at this optimal pH. Moreover, our results strongly suggest that the relatively high activity of electron source at this optimal pH was most likely a result of decreasing the ratio of bisulfite to sulfite in the cell cultures, since the toxicity of sulfite on PSII complex is lower than that of bisulfite. Subsequently, this suggestion is corroborated by the result that treatment with a combination of bisulfite and sulfite further enhanced the yield of H_2_ photoproduction in *C. reinhardtii* cells. During treatment with this combination of bisulfite and sulfite for H_2_ photoproduction, we unexpectedly found that sulfite addition can remove O_2_, activate H_2_ase and increase H_2_ photoproduction, just like bisulfite addition.

## Methods

### Culture conditions

*Chlamydomonas reinhardtii* cells (CC-503 strain) were cultured at 25 °C in Tris–acetate–phosphate (TAP) medium [46]. The medium was buffered with Tris–HCl (20 mM; pH 7.3), bubbled with air under continuous illumination with cool-white fluorescence lamps (40 μE m^−2^ s^−1^), and inoculated with approximately 8.1 × 10^4^ cells mL^−1^ of *C. reinhardtii* (inoculum size 1%).

### pH, NaHSO_3_ and Na_2_SO_3_ treatments

*Chlamydomonas reinhardtii* cells were cultured in 0.5 L of TAP medium for 2 days (*A*_750_ = 0.8–1.0). Subsequently, different pH values were modulated by adding 5 mM of NaOH or HCl to the cultures incubated at corresponding buffers. Next, a fixed volume of cells containing 300 µg chlorophyll (Chl) was transferred to 60-mL serum bottles (30 mL head space and 30 mL cells) with rubber seals. After a 36-h pre-culture stage, 13 mM of NaHSO_3_ or a combination of 7 mM NaHSO_3_ and 6 mM Na_2_SO_3_ or 25 mM of Na_2_SO_3_ was added to the serum bottles. The cells were then grown under continuous illumination (200 μE m^−2^ s^−1^) to induce the production of H_2_.

### Monitoring H_2_ photoproduction

At predetermined time intervals, 200 µL of gas samples were withdrawn from the bottles with a gas-tight syringe and injected into a gas chromatograph (Agilent 7890A; Agilent Technologies Inc., USA) with a thermal conductivity detector for determining the concentrations of H_2_, O_2_, and N_2_ simultaneously. The column was a molecular sieve column (type 5Å; 2 m × 1/8 mm). Argon was used as the carrier gas.

### H_2_ase activity assay

In vivo and in vitro H_2_ase activity was monitored using a previously described method [20, 22, 29, 38] with slight modifications. In brief, 1-mL cell suspension samples were withdrawn anaerobically from the 60-mL serum bottles at designated times (see Fig. 2b, c; Additional file 1: Figure S3b, c) and then injected into 10-mL glass vials. To measure in vivo H_2_ase activity, the samples were immediately exposed to argon gas for 1 min to eliminate the inhibitory effect of O_2_ on H_2_ase. The samples were then placed in a 25 °C water bath for 1 h and shaken continuously (150 rpm) while exposed to constant light (200 μE m^−2^ s^−1^ intensity). To measure in vitro H_2_ase activity, we used vials containing 1 mL of 10 mM oxidized methyl viologen prepared in O_2_-free 50 mM Tris buffer (for pH 7.1–9.0) and 0.2% (*w*/*v*) Triton X-100. The reaction was started when methyl viologen was reduced by the addition of 100 µL of 100 mM anaerobic sodium dithionite in 0.03 N NaOH. This assay was performed at 37 °C in the dark for 20 min. We determined the amount of H_2_ produced in the headspace of the glass vial by gas chromatography, and the rate of H_2_ production was calculated based on the total Chl content in the glass vial, unless otherwise indicated.

### Dissolved oxygen measurement

A dissolved oxygen (DO) meter (Orion Star A213, Thermo Scientific, USA) was used to monitor the DO attenuation process after the addition of NaHSO_3_ to the cultures of *C. reinhardtii* at different pH values. The DO meter was corrected before each measurement. The DO meter probe was placed in the middle of the cultures and the data recorded at different times.

### Chl fluorescence and P700 analysis

The Chl fluorescence yields at a steady-state of electron transport were measured at room temperature with a Dual-PAM-100 monitoring system (Walz, Effeltrich, Germany) equipped with an ED-101US/MD unit [47, 48]. Minimal fluorescence at open PSII centers in the dark-adapted state (*F*_o_) was excited by a weak measuring light (650 nm) at a photon flux density of 0.05 to 0.15 μE m^−2^ s^−1^. A saturating pulse of red light (600-ms, 10,000 μE m^−2^ s^−1^) was applied to determine the maximal fluorescence at closed PSII centers in the dark-adapted state (*F*_m_). Maximal quantum yield of PSII (*F*_v_/*F*_m_) was evaluated as (*F*_m_ − *F*_o_)/*F*_m_ [49, 50]. The redox state of P700 was measured with the aforementioned Dual-PAM-100 fluorometer. The P700 was oxidized by far-red light from a photodiode (FR-102, Walz, Effeltrich, Germany) for 30 s, and then the kinetics of re-reduction of P700^+^ in the dark was monitored.

### Oxygen exchange

The production of photosynthetic O_2_ in intact *C. reinhardtii* cells was measured at 25 °C by monitoring the evolution of O_2_ with a Clark-type oxygen electrode (Hansatech Instruments, Kings Lynn, UK). Oxygen production by photosynthesis was measured in the presence of 10 mM NaHCO_3_. The intensity of light used for the measurements of O_2_ evolution activity was 1000 μE m^−2^ s^−1^.

Light-induced oxygen uptake in intact cells of *C. reinhardtii* was determined at 25 °C by the oxygen consumption using an aforementioned Clark-type oxygen electrode as described previously [21, 35, 36]. The intact cells of *C. reinhardtii* were pre-illuminated by red light (> 630 nm; 1000 μE m^−2^ s^−1^) for 2 min before illumination by the same light to measure the oxygen consumption rate. The oxygen consumption was started by addition of bisulfite to the cell cultures and the photooxidation level of bisulfite was calculated as the rate of oxygen consumption in light minus that in darkness.

### Crude protein extract and immunoblotting analysis

To obtain crude protein extracts of *C. reinhardtii*, cells were harvested by centrifugation at 5000*g* for 2 min at 4 °C. The cell pellet was resuspended in 50 mM Tris–HCl, pH 8.3 plus 1% Triton X-100 and the suspension was shaken for 30 min in the dark [51]. Subsequently, the homogenate was centrifuged at 5000*g* for 5 min at 4 °C to remove unbroken cells and debris. A 200-µL aliquot of the supernatant was mixed with 100 µL of protein lysis buffer [2 M urea; 0.5 M Tris–HCl, pH 8.0; 20% glycerol; 7.5% SDS; 2% (*v*/*v*) mercaptoethanol; 0.05% (*w*/*v*) bromphenol blue] and heated for 5 min at 95 °C. After centrifugation at 12,000*g* for 10 min at 4 °C, the crude protein extracts were loaded onto the gels. SDS–PAGE was conducted as described before [52] using a 10% (*w*/*v*) separating gel. Immunoblotting was performed with an enhanced chemiluminescence (ECL) assay kit (Amersham Pharmacia Biotech) according to the manufacturer’s protocol. Antibody against Rubisco large subunit (RbcL) of *C. reinhardtii* was kindly provided by Dr. Lili Xu (College of Life Sciences, Shanghai Normal University).

### Superoxide anion assay

Superoxide anion was measured according to the instruction of superoxide anion kit (Nanjing Jiancheng Bioengineering Institute, China). The superoxide anion radicals in the assay kit were generated by the xanthine/xanthine oxidase reaction system to form a colored compound with a peak absorbance at 550 nm.

### Sulfate measurement

Sulfate concentration was measured using a previously described method [21, 53] with some modifications. In brief, 1-mL cell suspension samples treated by Na_2_SO_3_ or not were withdrawn from the 60-ml serum bottles at 0 or 12 h (Additional file 1: Figure S3b) and were supersonically disrupted with a power of about 100 W by six repetitions of a 20-s pulse followed by 3-min incubation on ice. The homogenate was centrifuged at 12,000*g* for 5 min at 4 °C to remove unbroken cells and debris. Prior to measurement, the supernatant was filtered through a 0.22-µm filter. Subsequently, SO_4_^2−^ in the supernatant was determined using an ICS-5000 ion chromatograph (Dionex) equipped with an IonPac AG19 guard column (4 mm × 50 mm) and an IonPac AS19 separation column (4 mm × 250 mm), connected with a conductivity detector (Dionex).

## Supplementary information

**Additional file 1: Figure S1.** Treatment with optimal pH decreases the photooxidation (a) and superoxide anion (b) levels. **Figure S2.** Comparison of decreased dissolved oxygen (DO) levels caused by addition of 7 mM NaHSO_3_ alone and a combination of 7 mM NaHSO_3_ and 6 mM Na_2_SO_3_ to the serum bottles. **Figure S3.** Treatment with Na_2_SO_3_ significantly increases the yield of H_2_ photoproduction in *C. reinhardtii*. **Table S1.** Concentrations of SO_4_^2−^ in Na_2_SO_3_-treated cultures of *C. reinhardtii* for different times.

## Data Availability

All data generated or analyzed during this study are included in this published article and Additional file 1.

## Abbreviations

AA: Antimycin A
CBB: Coomassie brilliant blue
*C. reinhardtii*: *Chlamydomonas reinhardtii*
*F*_v_/*F*_m_: Maximum quantum yield of photosystem II
GA: Glycolaldehyde
Lin: Lincomycin
PSI CET: Cyclic electron transport around photosystem I
RbcL: Rubisco large subunit
TAP: Tris–acetate–phosphate
