# Supplementary material for: Mechanistic insights into pH-dependent H2 photoproduction in bisulfite-treated Chlamydomonas cells
Source: Biotechnol Biofuels. 2020 Apr 6;13:64. doi: 10.1186/s13068-020-01704-0 (PMC7132995; doi:10.1186/s13068-020-01704-0)
Supplement: Supplementary file 1 — Additional file 1: Figure S1. Treatment with optimal pH decreases the photooxidation (a) and superoxide anion (b) levels. Figure S2. Comparison of decreased dissolved oxygen (DO) levels caused by addition of 7 mM NaHSO3 alone and a combination of 7 mM NaHSO3 and 6 mM Na2SO3 to the serum bottles. Figure S3. Treatment with Na2SO3 significantly increases the yield of H2 photoproduction in C. reinhardtii. Table S1. Concentrations of SO42− in Na2SO3-treated cultures of C. reinhardtii for different times. [file 13068_2020_1704_MOESM1_ESM.pdf]

Additional file 1

Mechanistic insights into pH-dependent H_2 photoproduction in bisulfite-treated *Chlamydomonas* cells

Lanzhen Wei[1], Baoqiang Fan[1], Jing Yi[1], Tianqun Xie, Kun Liu, Weimin Ma[*]

Shanghai Key Laboratory of Plant Molecular Sciences, College of Life Sciences, Shanghai Normal University, Guilin Road 100, Shanghai 200234, China

[1]These authors contributed equally to this work.

*Corresponding author: Weimin Ma, Tel.: +86 21 64321617, fax: +86 21 64322931, E-mail address: wma@shnu.edu.cn

Figure S1. Treatment with optimal pH decreases the photooxidation (a) and superoxide anion (b) levels.

Figure S2. Comparison of decreased dissolved oxygen (DO) levels caused by addition of 7 mM $NaHSO_3$ alone and a combination of 7 mM $NaHSO_3$ and 6 mM Na_2SO_3 to the serum bottles.

Figure S3. Treatment with Na_2SO_3 significantly increases the yield of H_2 photoproduction in *C. reinhardtii*.

Table S1. Concentrations of SO_4^{2-} in Na_2SO_3-treated cultures of *C. reinhardtii* for different times.

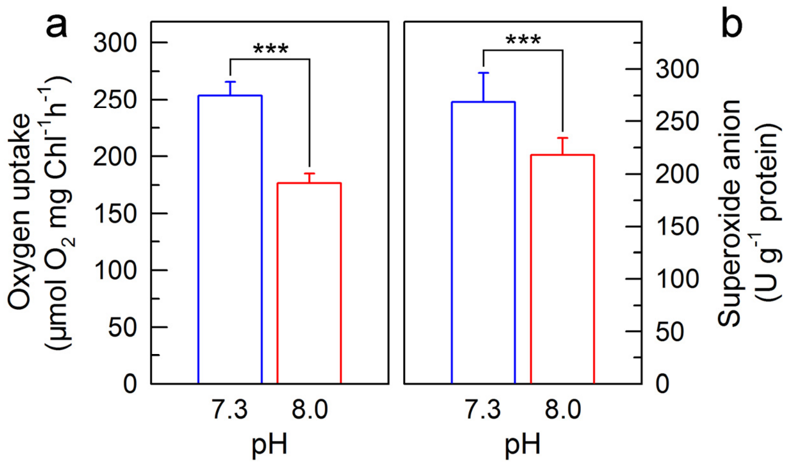

Figure S1. Treatment with optimal pH decreases the photooxidation (a) and superoxide anion (b) levels. Values are means ± SD (n = 5). ***$p < 0.001$.

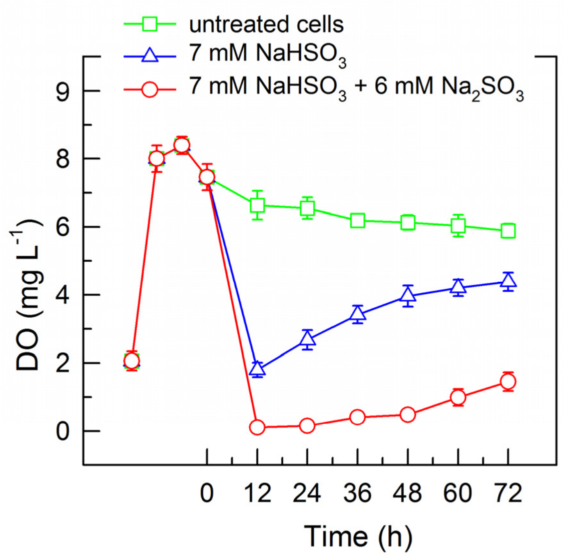

Figure S2. Comparison of decreased dissolved oxygen (DO) levels caused by addition of 7 mM $NaHSO_3$ alone and a combination of 7 mM $NaHSO_3$ and 6 mM Na_2SO_3 to the serum bottles. Values are means ± SD (n = 5).

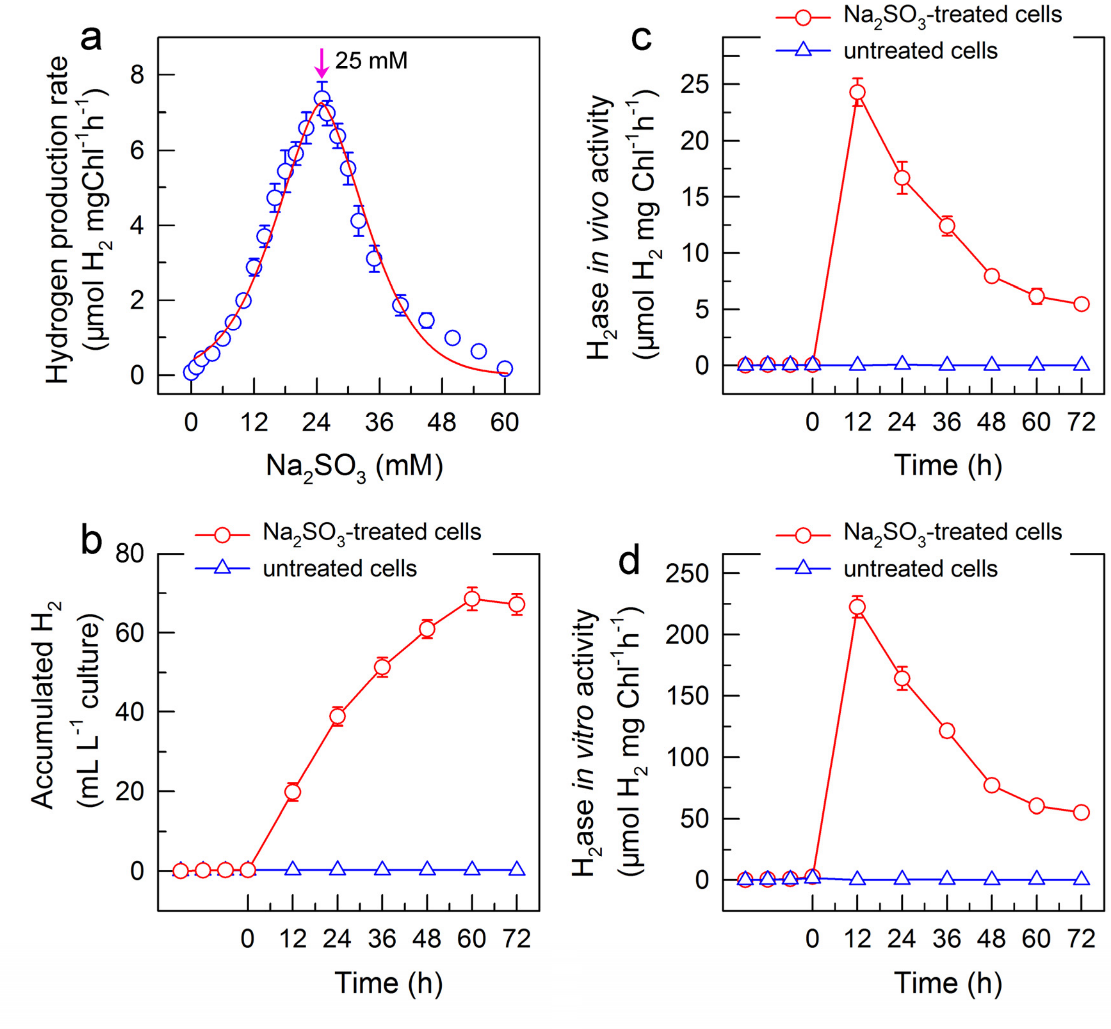

Figure S3. Treatment with Na_2SO_3 significantly increases the yield of H_2 photoproduction in *C. reinhardtii*. **a** Effects of treatment with different concentrations of Na_2SO_3 on H_2 photoproduction in *C. reinhardtii*. **b-d** Treatment with an optimal Na_2SO_3 (25 mM) significantly increases (b) H_2 photoproduction, and (c) *in vivo* and (d) *in vitro* H_2ase activity in *C. reinhardtii*. Values are means ± SD (*n* = 5).

Table S1. Concentrations of SO_4^{2-} in Na_2SO_3-treated cultures of *C. reinhardtii* for different times.

Time (h)	Na_2SO_3-treated cells (mg L^{-1})	Untreated cells (mg L^{-1})
0	64.8 ± 7.8	64.8 ± 7.8
12	1150.1 ± 33.5	60.5 ± 3.4

1 mL cell suspension samples treated by Na_2SO_3 or not were withdrawn from the 60 mL serum bottles at 0 h or 12 h (see Additional file 1: Figure S3b) and were supersonically disrupted. After centrifugation, SO_4^{2-} in the supernatant was analyzed by an ICS-5000 iron chromatograph. Values are means ± SD (n = 5).
